# Nurses’ Professional Development and Confidence Three Years Post-graduation: A Qualitative Study

**DOI:** 10.1177/23779608261477163

**Published:** 2026-08-18

**Authors:** Kari Toverud Jensen, Heidi Jerpseth

**Affiliations:** 1 Faculty of Health Sciences, 158935 Oslo Metropolitan University, Oslo, Norway

**Keywords:** nurses, professional confidence, professional development, qualitative study

## Abstract

**Introduction:**

The current nursing workforce shortage underscores the urgent need to understand nurses’ experiences and the factors they consider essential for retention. In this light there is growing interest in research that explores how nurses define their roles, knowledge, and professional development.

**Objectives:**

The aim of this study was to explore nurses’ work experiences three years after graduation and to identify what enabled their professional development and confidence.

**Methods:**

This research was part of a longitudinal qualitative study in which the same participants were interviewed three times over a span of four years. For this study, interviews were conducted three years after graduation, from March to April 2024. The researchers conducted individual semi-structured interviews with the same participants as in the previous two rounds. Data were analysed using Braun and Clarke’s Reflexive Thematic Analysis.

**Results:**

Two themes were constructed: professional insight and the meaning of collegial community. The main findings showed that the participants felt more confident both in caring for their patients and in how they expected and demanded to be taken seriously in interdisciplinary contexts. The participants often referred to their experiences and competence related to practical skills and to their ability to find solutions, as well as to their growing sense of authority associated with their profession as nurses.

**Conclusion:**

The findings indicated that professional insight and collegial community were intertwined, mutually constitutive components of professional self-esteem and development; each shaped the other, and together they influenced what nurses identified as enablers of their professional practice.

## Introduction

The current nursing workforce shortage underscores the urgent need to understand nurses’ experiences and the factors they consider essential for retention. In this light there is growing interest in research that explores how nurses define their roles, knowledge, and professional development (Buchan et al., 2022). This interest is partly driven by nurses’ role perceptions are linked to staff turnover and are often shaped by workload pressures and job dissatisfaction (Tamata & Mohammadnezhad, 2023). However, to the best of current knowledge, there is a limited number of studies that have examined nurses’ perspectives on their own nursing knowledge, as well as their professional confidence and development, at three years post-graduation.

### Review of Literature

Several studies have described how nurses perceive nursing knowledge and professional confidence from an early point in their careers. Rudman et al. (2020) found that around 12% of nurses in their study (N = 2474) reported high levels of burnout symptoms in at least one of the first three years of their careers. This research highlights the evolving nature of professional development, emphasising the importance of early career experiences for newly graduated nurses. Willman et al. (2022) explored the perceptions of nurses in an acute care hospital 18 months after they entered the profession. Their study revealed that these nurses, despite facing various challenges, developed a sense of “clarity and security in their nursing role”. This sense of professional stability was fostered by three key factors: “independence through personal effort and experience”, “well-functioning teamwork”, and “challenges in the work environment” (p. 3306).

It has been challenging to find a definition that fully encompasses the concept of professional confidence. However, in a concept analysis based on 21 articles, Holland et al. (2012) describe professional confidence as a dynamic, evolving personal belief held by a professional or a student. This includes an understanding of and a belief in the role, scope of practice, and significance of the profession, and is based on their capacity to competently fulfil these expectations, fostered through a process of affirming experiences (p.214). Some researchers have studied professional confidence in newly qualified nurses, such as Najafi and Nasiri (2023) who studied nurses’ experiences within the first year after graduation. They found that these nurses experienced disruptions in their professional life, challenging encounters, and a lack of knowledge that undermined their self-confidence. This is in line with Ortiz (2016) who explored the development of professional confidence in newly graduated nurses over the course of their first year. The research indicated that the nurses required exposure to both adverse and favourable experiences in order to effectively care for patients and manage critical situations during their initial year of practice.

Hakvoort et al. (2022) conducted a scoping review on factors influencing professional development in early nursing careers, and they found that professional identity in newly graduated nurses is shaped by knowledge acquisition, peer and mentor affirmation, and a sense of belonging (p. 4). However, they also found that nurses’ professional development needs vary by career stage, and their goals and strategies differ accordingly (p. 6). Rønnestad and Skovholt (2003) studied professional development in fields related to nursing, focusing on six phases of experience levels during the first five years after graduation. The fourth phase, known as the novice professional phase, encompasses the initial years following graduation, although individual paths vary. During this phase, the participants felt somewhat unprepared for the feelings of disillusionment and overwhelming emotions they experienced in their interactions with patients. They also developed an increased awareness of the complexity of their work and a deeper recognition of the importance of relationships in their clients’ progress. An interesting aspect of this phase was the shift from an earlier emphasis on skills to a more autonomous approach to learning and professional work (pp. 17-20). This phase model and its themes can be seen as providing a transferable roadmap for understanding nurses’ progression from graduation through to advanced practice.

As shown above, the available research (Hakvoort et al., 2022; Najafi & Nasiri, 2023; Ortiz, 2016; Rudman et al., 2020; Rønnestad & Skovholt, 2003; Tamata & Mohammadnezhad, 2023; Willman et al., 2022) highlights the complex interplay between personal effort, team dynamics, and external challenges in the process of becoming a professional. For newly graduated nurses, achieving a sense of clarity and security is essential for overcoming the inevitable challenges of the early stages of their careers. However, as they gain experience external aspects such as workplace culture, team collaboration, and institutional support become increasingly significant in sustaining their professional development and well-being. The evolving nature of these challenges calls for a more nuanced understanding of how professional development and confidence are fostered throughout a nursing career, emphasising the need for targeted support at various stages to ensure long-term success and satisfaction.

Based on these sources above, professional confidence is understood as a growing belief in one’s competence and in the value of the profession, and is reinforced by diverse experiences, autonomy, and teamwork, leading to role clarity and a sense of security. In addition, professional development is understood in this study as nurses’ experiences transitioning from a skills-centred mindset toward more autonomous, self-directed learning and practice, with individual paths varying as they become more aware of the complexity nursing roles and the centrality of professional relationships.

The research reported here is a part of a longitudinal qualitative study in which the same participants were interviewed three times over a four-year period (Jensen & Jerpseth, 2023; Jerpseth & Jensen, 2025). The first interview took place during the students’ third year of their bachelor’s degree; the second interview was conducted one year after they graduated as nurses. The purpose of the overarching project was to explore the participants’ understanding and perspectives of nursing knowledge over time. Qualitative longitudinal research refers to prospective or follow-up studies that are designed with two or more data collection sessions with the same participants over a specified timeframe (Snelgrove, 2014). In the first study, third-year bachelor’s degree students struggled to clarify and define their understanding of nursing knowledge, and participants regarded values as important and vital for becoming a professional nurse (Jensen & Jerpseth, 2023). In the second study, newly graduated nurses’ experiences related to practical readiness and the development of professional authority were examined (Jerpseth & Jensen, 2025). Those nurses who felt prepared demonstrated competence by combining their personal strengths with clinical judgment, enabling them to apply their skills and knowledge effectively in various situations. Participants perceived a discrepancy between their level of responsibility and professional authority, resulting in frustration and a sense of being professionally overshadowed, particularly by physicians (Jerpseth & Jensen, 2025).

### Aim

The aim of this study was to explore nurses’ work experiences three years after graduation and to identify what enabled their professional development and confidence.

## Methods

### Design

This study used an interpretive qualitative design to explore participants’ experiences. Individual semi-structured interviews were conducted, focused on nurses’ experiences of their professional roles and confidence. In addition, reflexive thematic analysis was chosen, conceptualising themes as interpreted patterns of meaning that were actively constructed by the researcher, with the researcher’s subjectivity used as a resource (Braun & Clarke, 2022). As part of a longitudinal qualitative study situated within a social constructionist epistemology, experience and knowledge were understood to be produced through social interaction and language (Burr, 2015).

### Sample

#### Recruitment and Participants

The same participants were interviewed three times over a four-year period. Participants were initially recruited via the Canvas learning platform, and nine students agreed to participate. These same individuals were interviewed throughout all three phases of the study. Initially, participants were informed that participation would involve three interviews, and an email was sent to confirm their continued willingness to take part in the study. No participants refused or dropped out.

#### Inclusion Criteria

The inclusion criteria for participants in the first study were that the participants had to be in their third year of a bachelor’s degree programme and be able to understand and speak Norwegian. In this study, an additional criterion was that participants were still working as nurses. No further exclusion criteria were applied. The initial interviews were conducted when the participants were third-year nursing students (Jensen & Jerpseth, 2023), the second round occurred one year after their graduation (Jerpseth & Jensen, 2025), and the final interviews in the project (this study) were conducted three years after graduation, in March–April 2024. The demographic characteristics of the participants are presented in Table 1.

**Table 1.** The Demographic Characteristics of the Participants

Participant	1	2	3	4	5	6	7	8	9
Male/Female	F	F	F	F	M	F	F	F	F
Age (years)	24	30	27	24	31	31	38	34	37
Workplace*	SC	SC	SC	SC	PC	PC	PC	SC	PC
Changed workplace	X	X	-	X	-	X	X	-	-
E/V*	-	E	E	-	E	E	V	V	V
​									

*PC = primary care, SC = secondary care.

**E = prior graduate qualification (e.g. economics, marketing, literary studies), V = prior vocational professions.

### Context

In the Norwegian education system, all nurses hold a publicly accredited bachelor’s degree. After completing their bachelor’s degree, all the participants worked as nurses. Five of the participants worked in secondary care (geriatrics, urology, paediatrics, emergency psychiatry) and four in primary care (home care, emergency room). Additionally, five of the participants had changed workplaces since completing their nursing education, one from secondary care to primary care while the other four changed workplaces within the same service level (Table 1).

### Data Collection

This study explored nurses’ work experiences three years after graduation and what enabled their professional development and confidence, thus warranting a qualitative approach.

The interview guide (Table 2) was developed as an extension and follow-up to the two previous articles, (Jensen & Jerpseth, 2023; Jerpseth & Jensen, 2025). Because both authors had actively participated in the previous two interviews, the researchers had already formed a picture of the participants prior to this third interview. Both researchers participated in the analysis process and discussed in advance the areas of interest and the need for additional knowledge. Nevertheless, the interview guide was structured in relatively broad thematic terms to ensure that participants’ experiences were clearly conveyed. Participants were interviewed via the digital platform Zoom. The interviews lasted from 28 to 69 minutes with an average duration of 40 minutes. All interviews were conducted by both authors, who are experienced qualitative researchers and have experiences from nurse education at the bachelors, masters, and PhD levels.

**Table 2.** Themes for the Semi-structured Interview Guide

Themes for the Semi-Structured Interview Guide
o Practical Procedures and Preparation
o Responsibility and Authority in Nursing
o Demands of the Profession vs. Knowledge Acquisition
o Perceiving One’s Own Role as a Nurse in (Interdisciplinary) Teamwork
o Professional Confidence
o Nursing Knowledge Today
o Defining Responsibility and Practice

### Transcription

Interviews were recorded using an application (nettskjema.uio.no) provided by the Oslo Metropolitan University. Although automated transcription was available, the first author listened to each recording and produced verbatim transcripts. Transcripts were de-identified by removing direct identifiers, including names, ages, and workplaces. Transcripts were not returned to participants for review or factual correction. An ongoing debate in qualitative research concerns returning transcripts to participants; in this study, transcripts were not returned. This decision is consistent with the recommendations of Braun and Clarke (2022, 2024).

### Data Analysis

In the data analysis we followed Braun and Clarke’s (2022) Reflexive Thematic Analysis (RTA) and their reporting guidelines (RTARG) to improve the methodological coherence and the reflexive openness (Braun & Clarke, 2024). In addition, a COREQ checklist (Tong et al., 2007) was attached.

The decision to use Braun and Clarke’s (2022) Reflexive Thematic Analysis was based on the understanding that themes were conducted through coding. The analytical process commenced during the interviews. Following each interview, reflections were discussed and tentative points of interest relevant to the study aim were identified, and consideration was given to whether specific issues should be emphasised in the next interview, based on the data and the theoretical preconceptions. This means that an abductive approach was adopted.

The researchers meticulously read and re-read all the interviews to familiarise it even better with the dataset and to gain a deeper understanding of each nurse’s experiences. The researchers then highlighted all the quotations deemed intriguing in relation to the study’s aim and compiled them into a word-matrix. Both researchers reviewed these quotes and conducted preliminary coding. Afterward the researchers convened to discuss the findings. The first author continued working on the data, suggesting the initial themes and creating a new word-matrix. These were deeply discussed and reflected upon several times by the researchers collectively before constructing two naming themes: professional insight and the meaning of collegial community (see Table 3). The two themes were constructed based on an abductive analysis of the data.

**Table 3.** Examples of the Data Analysis

Quotes	Coding	Generating initial themes	Naming themes
*The more you’ve worked, the more experience you’ve gained, and maybe the more confident you’ve become in yourself and your own knowledge. So it’s perhaps that confidence that you’re left with the most. I think there have been several situations where you’ve simply had to test that you know what you know, and then you’ve seen that you can do it and it’s perfectly fine*. (I:4)	Experience provides confidence in oneself and one’s own knowledge.	Self-confidence as nurses.	**Professional insight**
*Everything you do can be boiled down to what you learned in basic nursing, really.* … *you can kind of boil a lot down to what is, in a way, basic nursing. What it's about and that should be the basis regardless of what you do, and if you have those needs and those things covered then you are pretty “good”. It’s not perfect because there’s always more you can do if you have the opportunity, but*… (I:8)	It all boiled down to basic nursing knowledge	Perspective of nursing knowledge	
*I’ve been working for a few years now, so I feel like people know what my role is. When I say something, I usually feel heard*. (I:4)	Being heard and recognised	Collegial belonging and peer support	**The meaning of collegial community**
*I think I was a bit unprepared for the emotional aspect of nursing. So being allowed to vent, whether it’s crying or laughing at something. Or just*… *the worst emotions, and the best ones, really* (I:2)	Emotional belonging		
*It’s just so important to have a colleague who can come and look at the child in the middle of the night if you’re uncertain. It’s like, they can actually tell you that your assessments are completely correct. It’s reassuring to feel that you’re not going around being scared or doing something you’re unsure about.* (I:3).	Be reassured by a colleague so that you’re not walking around being scared.		

### Ethical Considerations

This study was conducted in accordance with the Declaration of Helsinki (WMA, 1964). The Norwegian Centre for Research Data granted approval for this study (project no. 845886). Participants had previously received an email detailing information about the study, and written consent was obtained for all three planned studies. Nonetheless, the researchers sent a follow-up email to verify their continued willingness to participate. In this communication, their rights to withdraw from the study at any point during the research process prior to the analysis phase were reiterated. The participants consented to the use of Zoom, a videotelephony software programme, for data collection. The application (nettskjema.uio.no) linked to Services for Sensitive Research Data to record the interviews was utilised. The audio files were secured with passwords, and access was restricted solely to the research team. The data were deconstructed and stored in compliance with the prevailing guidelines at the Oslo Metropolitan University and in accordance with the General Data Protection Regulation (GDPR).

### Findings

The main findings showed that the participants felt more confident both in caring for their patients and in how they expected and demanded to be taken seriously in interdisciplinary contexts after three years’ working experience as nurses. The participants often referred to their experiences and competence related to practical skills and to their ability to find solutions, as well as to their growing sense of authority associated with their profession as nurses. The researchers constructed two themes: 1) *Professional insight*, supported by two subthemes, and 2) *The meaning of collegial community*, supported by one sub-theme.

### Professional Insight

The findings under the theme of professional insight suggested two sub-themes: self-confidence as nurses and the perspective of nursing knowledge.

#### Self-Confidence as Nurses

Participants’ descriptions of feeling secure and having the courage to speak up were interpreted as evidence of increased self-confidence in their nursing role. One participant described a sense of security that stemmed from respectful communication with the physicians, as well as the ability to address and resolve instances of unprofessional behaviour in a constructive and professional manner.

… *if I get scolded by a physician, I respond by saying that I don't think it's acceptable to be spoken to in that manner. I also inform my colleagues if they call and receive a harsh response. I tell them, “No, you can demand this. You have the right to request an examination if the patient is unwell.” There can be time constraints and many reasons for responding in a certain way. The times I have said something like that, it has been very orderly, and even when I have reported incidents and informed those involved about filing a report, it has been handled in a way where we met, discussed, and resolved the issue.* (I:3).

In the same manner, another participant emphasised the importance of a shared understanding of patients throughout the treatment process, regardless of position, to ensure the best possible care and treatment. To be a part of the team and be heard and recognised as colleagues were emphasised as important by all of the participants. One of them noted that the feeling of being heard has evolved with more experience. Although she believes she might have been heard before, she did not realise that it was about that.

*It was probably like that when I started too. The only thing that has changed is, perhaps, that I now dare to use it. It's more about becoming a bit braver and more confident in what I observe myself.* (I:8)

Another participant described developing confidence and learning to trust herself in new ways after dealing with difficult situations that she managed either alone or with help from the emergency services.

*I believe that my* (self-confidence) *comes from having been in challenging situations, often alone as the nurse on duty. In those moments, I had to do my best and manage patients who might find the situation a bit difficult as well as manage their relatives. I found solutions or handled the situations well enough to learn that it worked fine. I also developed techniques, like knowing whom to call for support…… there's a lot of support available from the emergency services. Even if they can't always come out, at least you have someone to consult with if you're uncertain*. (I:6).

With experience, they felt increasingly heard and recognised, which fostered greater self-confidence and a more active role in patient care.

However, the hierarchical structure in decision-making posed an ongoing challenge for the nurses in their professional roles. This self-confidence also applied to their collaborations with physicians, with whom they felt more confident asserting themselves and expressing their professional insights and opinions. They emphasised the importance of continuity and of being included as an integral part of the patients’ treatment and care.

*We are allowed to be involved in the decision-making quite a lot or at least be part of the discussion.…I know, ultimately, that it is the physicians that are responsible if we make any changes. So, I feel like I am allowed to express my opinion and be heard on what I need.* (I:4).

This participant acknowledged the limitations of her role as a decision-maker. She noted that while she was allowed to be involved and express her opinion, she understood her position relative to the physician’s decision-making authority.

However, some of them described their own self-confidence as fragile because it depended on whether they managed tasks successfully or not. When faced with familiar tasks, they felt assured and capable, but new situations and tasks were often met with insecurity and hesitation. This sense of uncertainty was particularly apparent when they encountered situations that were outside their routine or required new skills. Despite their growing experience, they acknowledged that their confidence remained vulnerable to the challenges posed by unfamiliar tasks, which highlighted the ongoing nature of professional development and the emotional ups and downs of nursing practice.

*In situations that I feel I have mastered, and tasks that I know I can handle, I have high confidence. But I can be a bit timid; there are some situations and tasks that I face where I think, “I have no idea how to approach this”.* (I:7).

The participants described their journey from newly graduated nurses to having three years of experience, which revealed a transformation from having felt intimidated by their responsibilities to having greater confidence as nurses. However, some were still fragile in their self-confidence, especially when they had to handle new tasks and situations.

#### Perspective of Nursing Knowledge

The findings highlighted the difficulty many nurses had in specifying their nursing knowledge, and they instead underscored the importance of practical experience and the crucial role of staying professionally updated. They also addressed the nurses’ responsibility for the patients’ physical and psychological needs. The essential function of coordination among various healthcare professionals to ensure comprehensive patient care was emphasised.

One of the participants reflected on her clinical journey and the breadth of her nursing knowledge, appreciating how her experiences had often shaped her understanding in ways that formal education cannot fully capture. She had observed that her colleagues read and perform procedures differently based on their practical and theoretical experiences, and this worked well.

*What I have learned and what I have read is so important, and you might become even more focused on what you've experienced before, compared to what you learned in school. I think* (nursing knowledge) *is very broad. Very comprehensive. Very individual. There's a big difference in what people have experienced.…. Sometimes you think you've read the procedure, and then the two of us, colleagues, are doing the same procedure, and we realise we've done it completely differently, and then you think, oh no, has something gone wrong? But in fact, both ways are fine.* (I:1)

One of the participants described the dual approach of learning and doing as essential in order to navigate the complexities and nuances of the nursing field.

*I don't think I would say I have knowledge about something in nursing if I haven't both read about it and experienced it, meaning putting it into practice* (I:2).

Some emphasised the importance of repetition and how their body memorises their reactions. They emphasised that repeated practice and exposure to similar situations had helped them manage new situations.

*It's about experience and repetition, if you want to put it that way. Things become ingrained in your muscle memory—you know you've encountered a similar situation before. You know how you react. You get to know yourself and understand how your body responds to stress, which is useful when you find yourself in a situation that might be somewhat frightening.* (I:8).

In reflecting on the relationship between experience, knowledge, and confidence, one participant noted:

*I feel that there are more situations now where I stand much more confidently than I did before*… *No, it is the combination of knowledge and experience that gives me this confidence* (I:4).

Another participant referred to her own experience as a patient and her reflections, which stemmed from this experience, on the importance of care in nursing. This experience emphasised for her that meticulous care was a crucial form of nursing knowledge. She also underlined that professional competence was not just about one’s initial education but also involved a commitment to ongoing learning and empathy.

*One thing I've been thinking a lot about lately, now that I've been a patient myself, is caring and how much it really matters. It has given me a new perspective on my job, like how important the little things are that sometimes I think I don’t have time for, or that I can’t be bothered with. It's just a pillowcase, but how much it can mean*… *At least for care, it’s an important part of nursing knowledge. I think being professionally updated is very important. You notice it when you yourself are hospitalised, that not everyone is professionally updated, and that’s really unfortunate.* (I:3).

In line with this, another participant stressed the importance of seeing and understanding most of the patients’ needs.

*I feel that I have more of a clinical perspective (than I had when I was newly graduated) that I see the whole patient and not just the specific issue, which might be more targeted towards aids and the operated hip. I try to talk and understand more about the patient's needs, considering both the psychological and physical aspects, and perhaps also looking at their nutrition.* (I:6).

There were differences in what the participants emphasised as the nurse’s main task as well as in their arguments for this choice. One of them described the role as being a coordinator:

*If there is information that needs to be coordinated, it is us* (nurses) *who do it. We assess the overall situation because the other agencies, such as general practitioners, occupational therapists, or physiotherapists, often see only part of the picture.* (I*:*5).

Even though many of the participants described nursing as challenging, with a huge workload and many responsibilities, they also valued it highly.

*I feel that a nurse should know a lot. There is much more responsibility than I thought. A much higher workload than I thought. But still, I love my profession* (I: 9).

The findings regarding the perspective of nursing revealed the complexity of nursing knowledge, emphasising the importance of practical experience, caring, and the variability in individual practices, as well as the necessity of staying professionally updated. A comprehensive approach was crucial, addressing both the physical and psychological needs of patients and ensuring coordination among healthcare professionals in provision of extensive patient care.

### The Meaning of Collegial Community

The findings showed that the participants emphasised the importance of being part of the ward community where they worked. This involved experiencing social belonging and interactions with colleagues. Most participants felt that they had found a place where they had the ability to grow professionally. Through the analysis, a sub-theme was constructed within the theme of the meaning of collegial community: collegial belonging and peer support.

#### Collegial Belonging and Peer Support

The participants emphasised the value of professional connections with interdisciplinary colleagues. They particularly highlighted the importance of close collaboration with other nurses, and shared experiences of being actively involved in patient care.

*We have a stable group of nurses. I also find that some of the more experienced doctors often include nurses in discussions. It makes me feel like we both have a lot to contribute, in a way* (I:3).

The participants described the importance of having colleagues who gave them space to vent their frustrations and emotions. As nurses, they met with people daily in various situations, with different diseases of varying severity, and they also interacted with different kinds of relatives. Managing their own feelings while meeting patients’ feelings, sorrows, and losses was something some of them were unprepared for. Having experienced and caring colleagues who encouraged them to share their frustrations and who believed that doing so made one a better nurse and better able to meet patients’ needs was seen as important.

…*”so when I come to the duty room and say that the patient is driving me completely mad”. And then you can go back to the patients and actually meet them where they are. Because you are a human being yourself. I think I was a bit unprepared for the emotional aspect of nursing. So, being allowed to vent, whether it's crying or laughing*… *Or just like*… *the worst feelings, and actually the best ones too. (I:*2*)*

Several participants highlighted that knowing how their colleagues worked, what their strengths were, and how they communicated made collaboration more efficient and less stressful. This knowledge made it easier to anticipate what might happen in different situations and to adapt quickly, which was especially important in high-pressure or challenging moments.

*What I always value highly are good, experienced colleagues*… *if you're unsure about something, ask a nurse or nursing assistant before asking a doctor.* … *that's made me so much better. I think I understand, and I also believe you become less worn down by the system when you have good people around you. It's the people who say, 'It's okay not to get everything done, someone will come after you'* (I:2)

Some participants reassured themselves that they possessed knowledge, even when they were uncertain. They were confident in their ability to handle new challenges, such as receiving a new patient, and they knew they could rely on their colleagues for support if needed.

*…., I have people to ask. I reassure myself that I know more than I think, in a way. This is going to go really well, though there will be some tough days getting to know the patient. Because it often comes down to feeling like there's a regular nurse who knows, who understands how they want all their medications and such things, so it's a lot about getting the time to familiarise …* (I:3).

Knowing each other’s working styles and communication habits made it easier to find solutions and to support each other in their tasks. Supporting one another during the challenging decision-making process seemed important for ensuring their safety and having someone to rely on.

*It's still a bit scary. I work nights with my regular colleague, and we have a routine where we ask each other questions. I can put the patient on hold and then consult with my colleague, and she does the same even though she has worked in emergency rooms for 12 years. If we are very unsure, we call the doctor or the emergency services*. (I:9)

The participants felt that their sense of belonging with their colleagues was important for their practice in the nursing profession. Additionally, the findings showed that being part of a team is crucial for the nurses’ sense of well-being at work. Being part of a common workforce was something many participants underlined as very important, as they could lean on experienced nurses for their knowledge and competencies, and they reported that this contributed to their resilience in meeting the challenges of their work.

## Discussion

The findings indicate that professional insight and collegial community are intertwined, mutually reinforcing components of professional self-confidence and development; each shapes the other, and together they influence what nurses identify as enablers of their professional practice.

It appears that the importance of a safe, collegial work environment that is inclusive and supportive lies in its ability to provide opportunities for professional growth and the development of professional knowledge. The subtheme “the perspective of nursing knowledge” may also be read as an enabling condition or resource for professional insight. The findings can also be understood as a process in which participants’ experiences shape their professional insight and understanding of the nursing profession, while their meaning-making is also influenced by engagement with a collegial community. This process may contribute to the development of their confidence and knowledge as nurses. In this study, “professional insight” refers to nurses’ professional growth developed through enhanced knowledge, experience, and reflection. This professional insight was intergraded gradually both as a prerequisite and development for professional self-confidence. Therefore, it is interesting to discuss the importance of collegial community and professional insight, and how these contribute to professional self-confidence and growth.

### Building Confidence and Professional Growth

The findings highlight the importance of being part of a team of colleagues, which is argued to enhance security and courage in the nursing role. This is achieved through respectful communication and the management of challenging situations, whether independently or collaboratively, such as with colleagues or with the emergency services. The collegial community emerged as one of the most important aspects for the nurses and highlights the importance of cooperation with experienced nurses and the value of being part of a team of colleagues. These findings suggest that after three years most of the nurses had found their places and experienced a sense of belonging, both in relation to the department they worked in and the nurses they worked with. In addition, nurses feeling heard and being taken seriously in the workplace might contribute to their overall sense of belonging and professional security. This is in line with Holland et al. (2012) who described professional confidence as dynamic, viewing it as a belief in one’s role as a nurse, which is promoted through a process of supporting experiences. However, Warshawsky et al. (2016) argue that a sense of belonging to people, places, and professions may influence individual decisions to remain in or leave a position or profession. In a time of nursing shortages, it is crucial to foster a workplace culture where a sense of belonging is integral, ensuring nurses feel supported and valued.

The importance of belonging has been documented in prior research as highly significant for nurses, although most of this research is only related to nursing students and newly graduated nurses (Ebert et al., 2019; Hunter & Cook, 2018; Metzger & Taggart, 2020; Patel et al., 2022). Many of the participants in this study emphasised the importance of being recognised as decisive contributors to both patient treatment and care and to the experience of being equals in the working community. Some participants described situations in which they advocated for nursing-related issues against what they referred to as “other professional authorities”, specifically physicians. The participants shared experiences of being taken seriously and being heard, but as some mentioned they were still well aware of their position in the professional hierarchy. Interestingly, Forbes and Evans’ (2022) and Jerpseth & Jensen (2025), show that newly graduated nurses struggle with stressors in communication with physicians. Findings from this study indicate that, after three years in their professional roles, nurses appear to have replaced earlier uncertainty with a sense of professional security and the courage to assert their views. This aligns with the findings of Holland et al. (2012), who describe professional confidence as a maturing personal belief and understanding of one’s role and of the profession’s significance, developed through an affirming process of experiences (p. 214).

To navigate in these relationships and assert their professional authority, nurses need solid knowledge, experiences and courage. It seems essential in the nurse-physician dynamic to be recognised as critical contributors to patient care and medical treatment. Based on this study, aspects such as professional trust and insight seem to be important for fostering a sense of community within the nursing profession.

When uncertainty and challenges are embraced, nurses can progressively develop professionalism and self-confidence; this process can build professional nursing knowledge through experience and enhance professional insight. Pursio et al. (2021), Skår (2009), and Thorne (2023) underscore the need for further research on how to strengthen the argumentative knowledge of newly graduated nurses. Enhancing their ability to advocate for patients’ rights and their own involvement in decision-making processes is recognised as an important area for improvement. The findings presented here indicate that ongoing professional development and emotional resilience are crucial and play an integral role in patient care and in nurses’ ability to confidently collaborate with physicians. In this mutual cooperation, belonging may be viewed as both a prerequisite for professional growth and a consequence of such growth. The most important aspect is the evolution of self-confidence over time, driven by experience, repetition, and continuous professional growth, even when facing new challenges or interacting with other professionals. Patel et al. (2024) conducted a concept analysis of the sense of belonging in nursing and found that when a sense of belonging was present nurses showed improved confidence and self-esteem and were more motivated to learn. They also described the sense of belonging as a fundamental driving force within social groups that is critical to the nursing profession. In addition, Allen et al. (2021) conducted a review of the conceptual issues of belonging. They identified four interrelated core components of belonging – competencies, opportunities, motivations, and perceptions – that can increase people’s sense of belonging at both the collective and individual levels. The findings of the study appear to address some of these components, as reflected in the way the participants generally referred to their own professional development. He et al. (2024) explored the factors that influence the development of nursing professionalism, among which promoting the growth of nursing professionalism through self-activation and overcoming challenges was seen as important.

The findings might be understood as what Wenger-Trayner and Wenger-Trayner (2020) refer to as a social learning space, which is generated by three characteristics of participation – caring to make a difference, engaging uncertainty, and paying attention. In the understanding of the characteristics, “caring to make a difference” specifies that participants want to increase their ability to do something, which in this study might be to increase their body of knowledge in nursing, and through this affect their working situation in a way they care about. They define the engagement of uncertainty as the tension between wanting to make a difference and finding a clear path, as expressed through various forms, thus fostering social learning through mutual participation. Paying attention is the third generator of a social learning space, and this implies an active stance that is integral from the first interaction and encompasses both attentive listening and proactive feedback-seeking.

### Strengths and Limitation – Reflections of the Work

For the participants included in this study, by three years post-graduation, they had established their place within a professional collegium and as members of inter-professional teams. They possess a clear understanding of their professional position relative to other professions and exhibit both the courage and knowledge required to assert their professional voices. For educators in nursing education, it is essential to have a thorough understanding of this crucial knowledge – that becoming a professional nurse and experiencing professional confidence takes time – when supervising nursing students. Emphasising the importance of time is imperative because this appears to foster professional confidence in nurses and serves to bridge the gap between theoretical knowledge and practical experience, thus enables professional development.

The participants of this study had been involved in the overarching project for four years. None of them had left the nursing profession, and overall, they conveyed a very positive impression of their work situation and their choice to pursue a career in nursing. This may be because they represented a selected group who sought participation in this project and may thus have had a greater interest in research and the nursing profession. It might also be the result of few of them having changed workplaces outside their professional specialisation. Of the five who changed workplaces, only one changed their service level. The feeling of complexity in their work is now met with recognition and an increased awareness (Rønnestad & Skovholt, 2003) compared to what they experienced as newly graduated nurses.

This study constitutes the final segment of a qualitative longitudinal study that was conducted over several years. Repeated engagement with the same participants on three occasions, along with reflections spanning from the project’s inception to the final interview may have influenced the analytical perspective. In the methodology section, preconceptions and reflections throughout the analysis are detailed. The article, particularly the analysis was also deliberated on with critical academic colleagues within the Educational Research group to which the authors belong. This collaboration has provided us with enhanced insights and ensured a rigorous evaluation of the project.

Throughout the research process, the researchers reflexively examined their decisions, asking questions such as “How do we know what we know?” and “Could this knowledge be understood differently?” A further limitation is the small sample size, and participants may have had a particular interest in the topic. Nevertheless, given the qualitative design, the focus is on the depth of these participants’ experiences.

### Implications for Practice

A pedagogical implication of this study is the recognition that nurses’ professionalism and self-confidence develop over time. This should be reflected in learning objectives and outcomes; in structured reflection before, during, and after clinical practice; and in supervision – especially in the final phase of nursing education, when students may be more receptive to what participation in the workforce entails.

### Recommendations for Further Research

Further research on this project will involve conducting a secondary data analysis in which the dataset from all three studies is re-examined. This approach will allow for a better understanding of how the individual processes of professional development and confidence unfold over time. This knowledge might be crucial for the further development of nursing education and for understanding how newly qualified nurses are integrated into clinical settings. Data from the longitudinal study, which this paper is a part, can be used to develop a survey with more participants, thereby providing a broader understanding of how nurses’ competence develops over time.

## Conclusions

The findings highlight that, after three years in nursing, the context of a collegial community is essential for professional growth, self-confidence, and emotional resilience. The participants have developed new forms of role complexity, consolidated their professional identities, and experienced shifts in agency. They have also strengthened their argumentation skills and experiential knowledge in nursing. This development can also be understood as changes in collegial integration that extend and update existing knowledge. Aspects such as feeling heard and valued enhance their ability to collaborate, advocate for patients, and navigate professional hierarchies. By cultivating supportive environments that encourage learning and trust, nurses can transform uncertainty into self-assurance, ensuring better patient care and professional fulfilment.

## Data Availability

The interviews and the analysis are available in Norwegian language. The data is cited in Table 3. Examples of analysis, and in the Findings paragraph.*
